# Anti-*Leishmania amazonensis* Activity of Morolic Acid, a Pentacyclic Triterpene with Effects on Innate Immune Response during Macrophage Infection

**DOI:** 10.3390/microorganisms12071392

**Published:** 2024-07-09

**Authors:** Vanessa Maria Rodrigues de Souza, Nicolle Barreira Maciel, Yasmim Alves Aires Machado, Julyanne Maria Saraiva de Sousa, Raiza Raianne Luz Rodrigues, Airton Lucas Sousa dos Santos, Maria Gabrielly Gonçalves da Silva, Ingrid Gracielle Martins da Silva, Karine Brenda Barros-Cordeiro, Sônia Nair Báo, Josean Fechine Tavares, Klinger Antonio da Franca Rodrigues

**Affiliations:** 1 Infectious Disease Laboratory, Campus Ministro Reis Velloso, Federal University Delta of Parnaiba, Parnaíba 64202-020, PI, Brazil; vanessa.rodriguess@ufdpar.edu.br (V.M.R.d.S.); b.nicollem@ufpi.edu.br (N.B.M.); machado03@ufpi.edu.br (Y.A.A.M.); jully.yanne@gmail.com (J.M.S.d.S.); raizzaraianneluz@gmail.com (R.R.L.R.); sousaairtonlucas@gmail.com (A.L.S.d.S.); mariagabriellyufdpar@gmail.com (M.G.G.d.S.); 2 Microscopy and Microanalysis Laboratory, Department of Cell Biology, Institute of Biological Sciences, University of Brasília, Brasília 70910-900, DF, Brazil; gracilias@gmail.com (I.G.M.d.S.); karine.brenda22@gmail.com (K.B.B.-C.); snbao@unb.br (S.N.B.); 3 Postgraduate Program in Natural Products and Synthetic Bioactive, Federal University of Paraíba, João Pessoa 58051-900, PB, Brazil; josean@ltf.ufpb.br

**Keywords:** *Leishmania amazonensis*, morolic acid, nitric oxide, reactive oxygen species, cytokines

## Abstract

Leishmaniasis is a group of infectious diseases transmitted to humans during vector bites and caused by protozoans of the genus *Leishmania*. Conventional therapies face challenges due to their serious side effects, prompting research into new anti-leishmania agents. In this context, we investigated the effectiveness of morolic acid, a pentacyclic triterpene, on *L. amazonensis* promastigotes and amastigotes. The present study employed the MTT assay, cytokine analysis using optEIATM kits, an H_2_DCFDA test, and nitric oxide dosage involving nitrite production and Griess reagent. Morolic acid inhibited promastigote and axenic amastigote growth forms at IC_50_ values of 1.13 µM and 2.74 µM, respectively. For cytotoxicity to macrophages and VERO cells, morolic acid obtained respective CC_50_ values of 68.61 µM and 82.94 µM. The compound causes damage to the parasite membrane, leading to cellular leakage. In the infection assay, there was a decrease in parasite load, resulting in a CI_50_ of 2.56 µM. This effect was associated with immunomodulatory activity, altering macrophage structural and cellular parasite elimination mechanisms. Morolic acid proved to be an effective and selective natural compound, making it a strong candidate for future in vivo studies in cutaneous leishmaniasis.

## 1. Introduction

Infectious diseases are the greatest cause of mortality and morbidity across the world, bringing negative effects to health but also to the economy by worsening socioeconomic vulnerability [1]. Infectious diseases can be caused by bacteria, viruses, fungi, or parasites, and are prevalent in countries where the vast majority of the population lives in poverty [2]. Further, as these diseases mainly affect underdeveloped countries, there is little investment in prevention or treatment.

According to the World Health Organization (WHO), leishmaniasis is among the leading causes of infectious disease deaths [3]. In Brazil, the disease has been transmitted in rural areas, and recently, not only in rural areas, but also in large municipalities and capitals [4]. 

The protozoans that cause leishmaniasis belong to the genus *Leishmania* and are transmitted to mammals through the blood meal bites of female sandflies infected with the parasite [5]. More than 20 species of *Leishmania* are infective to humans and other mammals.

The parasites develop in two stages: the promastigote, with an external flagellum and entry into the vector’s digestive system, and the amastigote form, with an internalized flagellum, this being an obligatory intracellular form, found in mammalian monocytic phagocytic system cells [6,7]. Leishmaniasis manifests clinically in two forms: cutaneous leishmaniasis (CL) and visceral leishmaniasis (VL). The latter represents the most severe form with the highest mortality rate, and when there is no effective treatment, it can be considered fatal [8,9,10]. Among the species that cause CL is *Leishmania (Leishmania) amazonensis*, with a high geographic distribution in Brazil. The species is responsible for a high proportion of cases evolving to severe anergic diffuse cutaneous leishmaniasis [5].

The classic treatment of leishmaniasis has been the same since 1945, and it uses pentavalent antimonials such as N-methyl glucamine antimoniate (Glucantime^®^) (Paris, France) and sodium stibogluconate (BP 88^®^) as first-line treatments. In refractory cases, second-line drugs are used, such as amphotericin B, pentamidine, paromomycin, and miltefosine [6]. However, these drugs present many disadvantages such as nephrotoxicity, hepatotoxicity, cardiotoxicity, pancreatitis, kidney dysfunction, high costs, and difficult administration. In addition, a high incidence of parasitic resistance already exists, which limits their use and effectiveness [7,8]. 

Therefore, due to treatment limitations, there is a need for new therapeutic options that are effective and safe. Natural compounds are a principal source of new biomolecules [9]. Many secondary metabolites from plants, such as triterpenes, are being tested due to their great diversity of active biomolecules [10]. Studies have reported the presence of anti-inflammatory [11], antimicrobial [12], antiadipogenic [13], antinociceptive [14], anticancer [15], and antileishmanial [16] properties. 

Morolic acid is an oleanane-type triterpene, derived from germanicol with a carboxyl group in C-28 [17]. It is still little discussed in the literature, considered only for other biological activities, with reports only of its anti-HIV, anti-HSV, anti-inflammatory, and antidiabetic activity [18,19,20]. The present study therefore sought to evaluate the effects of morolic acid on promastigotes and amastigotes, evolutionary forms of *L. amazonensis,* and its selectivity (SI) using mammalian cells such as J774.A1 macrophages and VERO cells.

## 2. Material and Methods

### 2.1. Reagents and Drugs

Schneider Insect Medium, Roswell Park Memorial Institute 1640 Medium (RPMI 1640), Griess reagent, 2μ,7μ-dichlorodihydrofluorescein diacetate (H_2_DCFDA), zymosan and neutral red (VN), tetrazoline 3-(4,5-dimethylthiazol-2yl)-2,5-diphenyl bromide (MTT), Entelan^®^ slide mounting medium, stabilized antibiotic solution (penicillin 10,000 U/mL; streptomycin 10 μg/mL), stabilized antimycotic antibiotic solution (penicillin 10,000 U/mL; streptomycin 10 μg/mL; amphotericin B 25 μg/mL), sodium cacodylate, and lead citrate were purchased from Sigma Aldrich (St. Louis, MO, USA). Amphotericin B was purchased from Cristália (São Paulo, SP, Brazil). Meglumine antimoniate (Glucantime^®^) was obtained from Aventis Pharma (São Paulo, SP, Brazil). Fetal beef serum (FBS) was purchased from Cultilab (São Paulo, SP, Brazil). Sodium dodecyl sulfate (SDS) was obtained from Mallinckrodt Chemicals (St. Louis, MO, USA). A panotype rapid staining kit was purchased from Laborclin (Curitiba, PR, Brazil). Sodium nitrite (NaNO_2_) was purchased from Vertec Fine Chemistry (Rio de Janeiro, RJ, Brazil). Glutaraldehyde, osmium tetroxide, uranyl acetate, and Spurr resin were purchased from Electron Microscopy Sciences, Hatfield, PA, USA.

### 2.2. Morolic Acid

**Figure 1:**
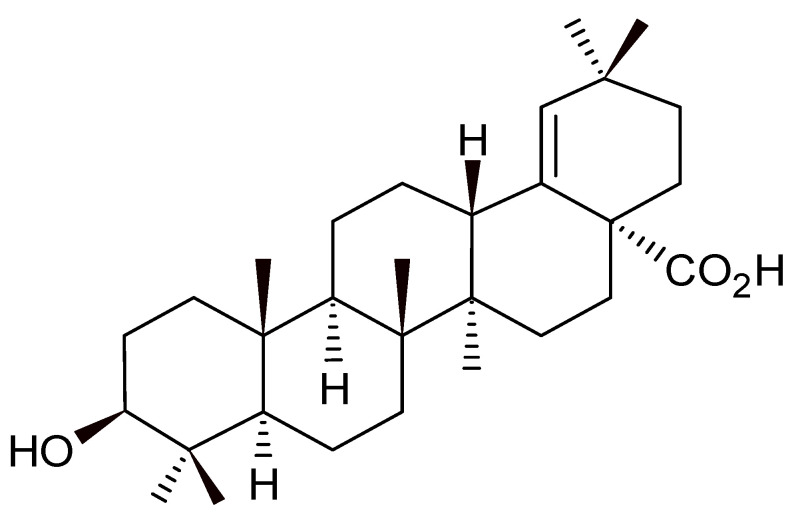
Chemical structure of morolic acid.

Figure 1 presents the chemical structure of morolic acid, C_30_H_48_O_3_, 97% purity, acquired from BioCrick. The compound was solubilized in dimethyl sulfoxide (DMSO), and the solutions were diluted until reaching the desired concentrations, not exceeding 0.5% DMSO.

### 2.3. Parasite Cultivation

The species *Leishmania* (*Leishmania*) *amazonensis* (IFLA/BR/67/PH8) was maintained in promastigote form at a temperature of 26 °C in a biochemical oxygen demand incubator (BOD) (Eletrolab EL202, São Paulo, Brazil) and in complete Schneider medium (20% heat-inactivated fetal bovine serum (FBS), and 1% antibiotic solution). The *L.* (*L.*) *amazonensis* axenic amastigote forms were obtained through the differentiation of infective promastigote forms using Schneider medium supplemented with 5% FBS, pH 4.6, at a temperature of 32 °C. Parasite growth was observed daily and, if necessary, weekly repeats were performed [21].

### 2.4. Murine Macrophages and VERO Lineage Cells

Murine J774A.1 lineage macrophages and VERO CCL 81 cells were grown in 750 cm^2^ cell culture flasks (Corning Glass Workers, New York, NY, USA) containing complete RPMI medium (10% SFB and 1% stabilized anti-mycotic–antibiotic solution), incubated at 37 °C and 5% CO_2_. The cells were observed on all days and replicates were always conducted after the cells reached confluence, around 72 to 96 h [22].

### 2.5. Antileishmanial Activity of Morolic Acid on Axenic Strains of L. (L.) amazonensis—Promastigotes and Amastigotes

The inhibition effects of morolic acid on growth for axenic forms were evaluated using the MTT colorimetric method. Promastigote forms or axenic amastigotes in an exponential growth phase were cultured in 96-well plates with complete Schneider’s medium, at 1 × 10^6^ parasites/mL. Serial concentrations of morolic acid (1.56–100 µM) and the reference drugs meglumine antimoniate (200–40,000 µM) and amphotericin B (0.031 to 2 µM) were added. The plates were incubated for 72 h under a BOD at 26 °C to prepare promastigotes and 32 °C to prepare amastigotes. When ready, 10 μL of MTT (5 mg/mL) was added to all wells (with the exception of the blank) with an incubation for 4 h. Subsequently, 50 μL of SDS solution (dodecyl sodium sulfate) at 10% was added to dissolve the formazan crystals, and a spectrophotometric reading was then performed (Biotek model ELx800, Winooski, VT, USA) at 540 nm [22]. Schneider medium with 0.5% DMSO was used, considering a 0% growth inhibition for the negative control.

### 2.6. Cytotoxicity of Morolic Acid on J774A.1 Macrophages and VERO CCL 81 Cells

The cytotoxicity assay was performed in 96-well plates using MTT for cell viability observation. Plates containing 100 μL of complete RPMI medium and approximately 5 × 10^5^ J774A.1 macrophages or VERO CCL 81 cells were incubated for 4 h at 37 °C and 5% CO_2_ for cell adhesion to occur. The board was then washed with phosphate-buffered saline (PBS—145 mM NaCl, 9 mM Na_2_HPO_4_, 1 mM Na_2_HPO_4_, pH 7.4) to remove non-adhering cells, and then added to RPMI medium together with morolic acid in concentrations of 1.56–200 μM. As a positive control, the reference drugs meglumine antimoniate (200–40,000 µM) and amphotericin B (0.031–2 µM) were used, and as a negative control, complete RPMI 1640 medium at 0.5% DMSO was used (considering 100% cell viability). After 72 h of incubation at 37 °C and 5% CO_2_, 10 μL of MTT was added and incubated for another 4 h at room temperature. The medium was then removed, and 100 μL of DMSO was added to dilute the formazan crystals created by the MTT [23]. Finally, the plate was taken to be read at 540 nm in a (Biotek model Elx800) spectrophotometer. 

### 2.7. Ultrastructural Analysis

To carry out the tests, promastigote forms of *L.* (*L.*) *amazonensis* in the logarithmic growth rate (1 × 10^6^) were incubated with the IC_50_ of morolic acid and stored for 24 h in a BOD at 26 °C. Washes were then performed with PBS, fixed with Karnovsky (2% paraformaldehyde and 2% glutaraldehyde) in sodium cacodylate buffer (0.1 M, pH 7.2) for 4 h, and then washed in 0.1 M sodium cacodylate buffer, pH 7.2, and post-fixed for 1 h in a solution with 1% OsO_4_, 0.8% potassium ferricyanide, and 5 mM CaCl_2_ in 0.1 M sodium cacodylate buffer, pH 7.2. Then, the samples were dehydrated using increasing concentrations of acetone (30 to 100%). For analyses in the transmission electron microscope, the material was embedded in Spurr resin. An ultramicrotome (Leica EM, Wetzlar, Germany) was used to obtain ultra-thin sections, and after this, the sections were contrasted with uranyl acetate and lead citrate for analysis in a Jeol JEM 1011 transmission electron microscope (Jeol, Tokyo, Japan). For observation in scanning electron microscopy after dehydration, the material was dried to the critical point (Balzers CPD 030, Schalksmühle, Germany) with CO_2_, metallized (Leica EM SCD 550, Wetzlar, Germany) with gold, and a Jeol JSM-7001F scanning electron microscope (Jeol, Tokyo, Japan) was used for the analysis [24,25].

### 2.8. In Vitro Activity of Morolic Acid against Intramacrophageal Amastigotes

Macrophages were added to 24-well culture plates containing complete RPMI medium and sterile 13 mm coverslips, 1 × 10^5^ J774A.1, and for cell adhesion incubated at 37 °C and 5% CO_2_ for 3 h. The medium was then removed, and three washes with PBS at 37 °C to remove non-adherent cells were performed. New medium, together with metacyclic promastigote forms in a proportion of 10 promastigotes/macrophage, was then added to the wells for infection and then incubated for another 4 h at 37 °C and 5% of CO_2_. The medium was then removed again and the plate washed with PBS to remove non-internalized amastigotes. About 1 mL of complete RPMI medium and varying concentrations of morolic acid (1.56–12.5 μM), amphotericin B (0.031–2 μM), and meglumine antimoniate (200–40,000 μM) were added to the plate and left under incubation for 72 h. Subsequently, the coverslips were removed, stained with a quick panoptic kit, and mounted with Entelan^®^. In each coverslip, 300 macrophages were counted, together with the number of internalized amastigotes through optical microscopy at 1000× magnification. For the negative control, complete RPMI medium with 0.5% DMSO was used [21]. At the end of the macrophage infection assay, the supernatant was stored at −20 °C for the subsequent evaluation of NO production.

### 2.9. Production of Reactive Oxygen Species (ROS)

The evaluation of ROS levels in J774A.1 macrophages infected with *L.* (*L.*) *amazonensis* and treated with morolic acid was performed using the H_2_DCFDA test. Macrophages were incubated in 96-well plates with complete RPMI medium for 3 h at 37 °C, and 5% CO_2_ for adherence. These were then infected with *L.* (*L.*) *amazonensis* promastigotes, at a ratio of 10 promastigotes per macrophage (10/1) and incubated for 4 h. Morolic acid was then added in concentrations of 1.56 to 12.5 μM for 72 h. Then 10 μL of H_2_DCFDA was added for a final concentration of 20 μM and incubated for 30 min in the dark at 37 °C. A spectrofluorometer (FLx800) was used to read the fluorescence intensity, using 485 nm of excitation and 528 nm of emission. Antimycin A (5 μM) was used as the positive control for ROS generation [22].

### 2.10. Production of Nitric Oxide by Infected Macrophages

The measurement of NO production (using macrophage infection supernatant) was performed by the production of nitrite with Griess reagent. About 100 µL of infection supernatant was added to a 96-well plate. In parallel, serial concentrations of NaNO_2_ in RPMI medium were also added to the plate for interpolation of the standard curve. Griess reagent (1% Sulfanilamide in 10% (*v*/*v*) H_3_PO_4_ in Milli-Q^®^ water and in equal parts to 0.1% naphthylenediamine in Milli-Q^®^ water Q^®^) was then added and incubated for 10 min at room temperature. At the end of the incubation, a reading was conducted on a plate reader at 540 nm [26]. The positive control was performed with *Escherichia coli* lipopolysaccharide at 2 μg/mL.

### 2.11. Assessment of Lysosomal Activity in Murine Macrophages 

The assessment of lysosomal activity was performed in 96-well plates using lineage macrophages J774A.1 (1 × 10^5^ /well) incubated together with morolic acid (12.5–1.56 μM) at 37 °C and 5% CO_2_ for 72 h. After this, 10 μL of PBS and 2% red neutral (VN) were added, with incubation in an incubator for another 30 min. Soon afterwards, the supernatant was aspirated and the plates washed 3× with PBS to remove excess VN. After this, for VN solubilization to occur inside the lysosomal vesicles, 100 μL of extraction solution (1% *v*/*v* glacial acetic acid and 50% *v*/*v* ethanol) dissolved in double-distilled water was added. Finally, the plates were placed under agitation in a Kline apparatus for 30 min, and then read in a spectrophotometer at 540 nm [27].

### 2.12. Phagocytic Capacity of Infected Macrophages

Morolic acid in serial concentrations (12.5 μM–1.56 μM) was added to plates together with macrophages (1 × 10^5^/well) and incubated at 37 °C and 5% CO_2_. At 72 h, 10 μL of VN-stained zymosan was added and then incubated for 30 min. The supernatant was then aspirated, and 100 μL of Baker’s solution (4% *v*/*v* formaldehyde, 2% *w*/*v* sodium chloride, and 1% *w*/*v* calcium acetate in distilled water) was added to stop zymosan phagocytic activity. To remove what was not phagocytized by the macrophages, the plate was washed with PBS. The plate extraction solution was added, and then solubilization was performed in a Kline shaker. The board was taken for reading on a spectrophotometer at 540 nm [7].

### 2.13. Assessment of Cytokine Production

According to Rodrigues et al., 2021 [27], to carry out cytokine evaluation, the supernatant saved after the J774A.1 macrophage infection assay was used to evaluate cytokine production. Analysis kits (optEIATM ELISA, Pharmingen, San Diego, CA, USA) were used following the manufacturer’s protocol to examine the release of cytokines (TNF-α, IL-1α, IL-6, IL-10, and IL-12), and standard recombinant cytokines were used to produce a curve. The reading was performed in a spectrophotometer at 450 nm.

### 2.14. Statistical Analysis

All assays were performed in triplicate and in 3 independent experiments. Differences between groups were analyzed by one-way ANOVA with post hoc Tukey testing, considering *p* < 0.05 as the maximum significance level. The values of the mean inhibitory concentration (IC_50_), mean effective concentration (EC_50_), and mean cytotoxicity concentration (CC_50_) were calculated (at 95% confidence intervals), using non-linear regression. To calculate the selectivity index (SI), the formula SI (selectivity index) = CC_50_/IC_50_ or EC_50_ was used.

## 3. Results

### 3.1. Evaluation of Antileishmanial Activity of Morolic Acid on Promastigotes and Axenic Amastigotes from L. (L.) amazonensis 

**Figure 2:**
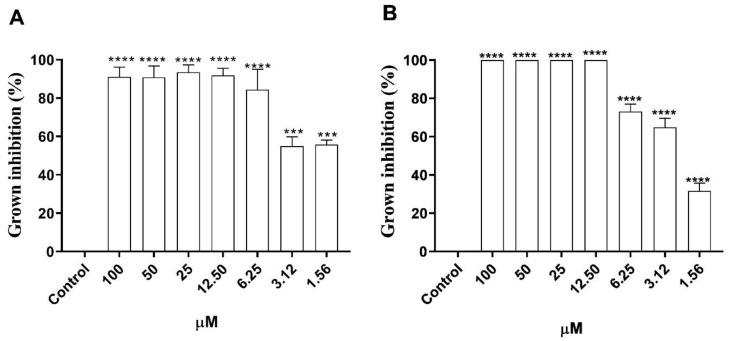
Effect of morolic acid on promastigotes and axenic amastigotes of *Leishmania (Leishmania) amazonensis*. Cultures in logarithmic growth phase (1 × 10^6^) were incubated at 26 °C (promastigotes) and 32 °C (amastigotes) for 72 h with morolic acid; and antileishmanial activity was evaluated using MTT colorimetric assay. Promastigote (**A**) and axenic amastigote (**B**) forms. Results represent mean ± standard error of three independent experiments performed in triplicate. (***) *p* < 0.001 vs. control; (****) *p* < 0.0001 vs. control.

Figure 2 shows the results regarding the inhibitory profile of morolic acid on promastigote forms of axenic amastigotes of *L.* (*L.*) *amazonensis*. Morolic acid caused a significant growth inhibition at all concentrations tested. At concentrations of 100 µM, 50 µM, 25 µM, and 12.5 µM, the compound was able to inhibit the growth of promastigote forms by 100%, followed by inhibitions of 83.3%, 57.85%, and 57.61% for concentrations of 6.25 µM, 3.12 µM, and 1.56 µM (Figure 2A), obtaining an IC_50_ of 1.13 ± 0.04 µM (Table 1). Regarding axenic amastigote forms, morolic acid inhibited the growth of these forms by 34.12%, 68.6%, and 76.4% at concentrations of 1.56 μM, 3.12 μM, and 6.25 μM, respectively (Figure 2B), presenting an EC_50_ of 2.74 ± 0.03 µM (Table 1). At concentrations of 100 μM, 50 μM, 25 μM, and 12.5 μM, the inhibition was 100% (Figure 2B).

### 3.2. Cytotoxicity of Morolic Acid on Murine Macrophages and VERO Cells 

**Figure 3:**
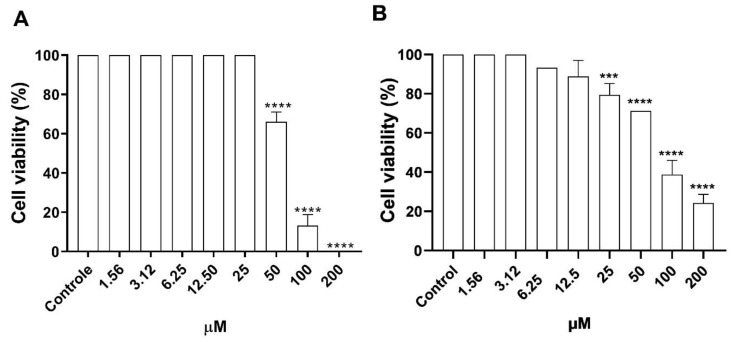
Cytotoxic effects of morolic acid on J774A.1 macrophages and VERO cells. Macrophages and Vero cells (5 × 10^5^) were incubated at 37 °C and 5% CO_2_ for 72 h with different concentrations of morolic acid. Cytotoxicity was assessed by MTT test. J774A.1 (**A**) macrophages. Vero cells (**B**). Results represent mean ± standard error of three independent experiments performed in triplicate. (***) *p* < 0.001 vs. control; (****) *p* < 0.0001 vs. control.

The cytotoxicity of morolic acid on mammalian cells was determined on J774A.1 lineage macrophages and VERO CCL 81 cells, and is shown in Figure 3. One may note that at concentrations of 1.56 μM, 3.12 μM, 6.25 μM, 12.5 μM, and 25 μM, the J774A.1 macrophages presented cell viability percentages of 100% when compared to the control. However, there was a respective reduction, as cell viability decreased to 65%, 15%, and 0% at concentrations of 50, 100, and 200 μM (Figure 3A). A viability of 100% was observed in the VERO CCL 81 strain at concentrations of 1.56 μM and 3.12 μM. However, there was a decrease in the number of viable cells at concentrations of 6.25 μM, 12.5 μM, 25 μM, 50 μM, 100 μM, and 200 μM, presenting respective values of 93.2%, 88.8%, 79.4%, 71.2%, 38.8%, and 24.3% (Figure 3B). 

### 3.3. Assessment of Promastigote form Ultrastructures

**Figure 4:**
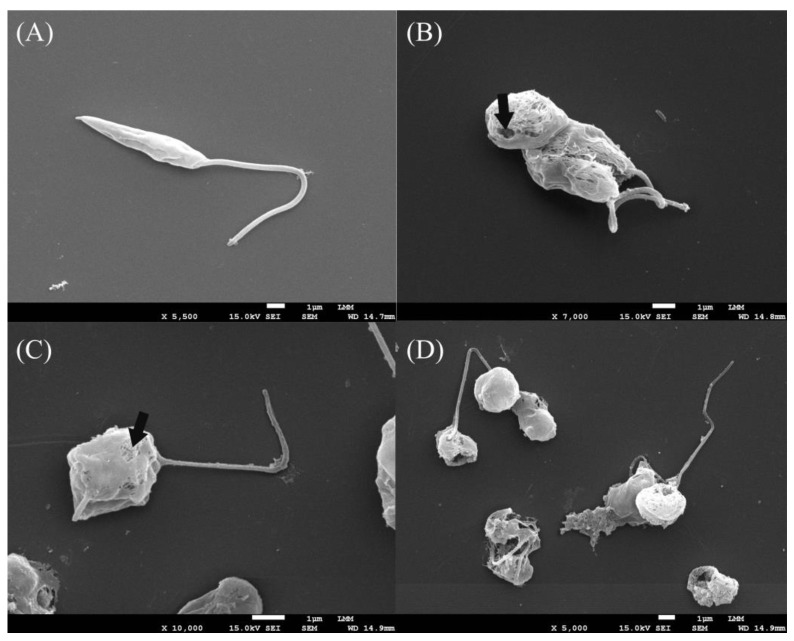
Ultrastructural analysis of promastigote forms of *Leishmania (Leishmania) amazonensis* treated with morolic acid, observed by scanning electron microscopy (SEM). The promastigote forms were seeded in Schneider’s medium, treated with the mean inhibitory concentration (IC_50_) of morolic acid (1.13 µM) for 24 h and analyzed by SEM. (**A**)—negative control; (**B**–**D**)—promastigotes treated with morolic acid. Arrows represent membrane rupture.

To investigate and elucidate the changes caused in promastigote forms when treated with morolic acid, scanning electron microscopy (SEM) and transmission electron microscopy (TEM) were performed. Figure 4B–D present the analysis of promastigote forms of *L.* (*L.*) *amazonensis* treated with the IC_50_ of morolic acid, as evaluated by SEM. Significant changes were observed after treatment; the parasite presented a wrinkling and shortening of the cell body length, and numerous perforations and fragmentation of the plasma membrane led to the extravasation of contents into the extracellular medium (Figure 4B,C). In contrast, the negative control (Figure 4A) presented a normal morphology, with the presence of the flagellum, an elongated body, and an intact membrane.

**Figure 5:**
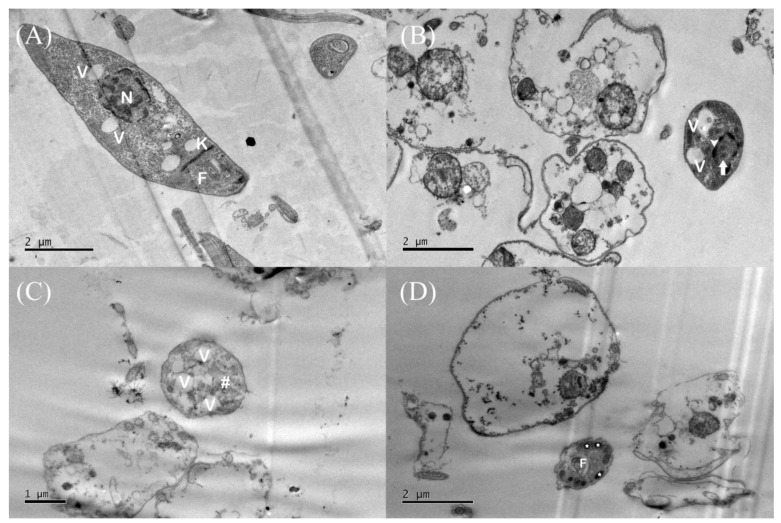
Ultrastructural analysis of promastigote forms of *Leishmania (Leishmania) amazonensis* treated with morolic acid, observed by transmission electron microscopy (TEM). The promastigote forms were seeded in Schneider’s medium, treated with the medium inhibitory concentration (IC_50_) of morolic acid (1.13 µM) for 24 h, and analyzed by TEM. (**A**)—negative control; (**B**–**D**)—promastigotes treated with morolic acid. (N) nucleus, (V) vacuoles, (K) kinetoplast, and (F) flagellar pouch. An increase in vacuoles is observed (**B**,**C**), the arrow indicates the compaction of chromatin and displacement of the nuclear envelope, the cytoplasm appears not very dense (#), evidenced in (**C**), and the appearance of lipid bodies is also noted (*) (**D**).

An ultrastructural analysis of *L.* (*L.*) *amazonensis* promastigotes treated with morolic acid was also performed using TEM. The negative control (untreated parasites) presented round nuclei, a regular kinetoplast morphology, and flagellar pockets (Figure 5A). The parasites treated with the IC_50_ of morolic acid presented cellular disorganization and an increase in the number of cytoplasmic vacuoles, pores in the membrane plasma, not very dense cytoplasm (Figure 5B,C), the presence of electron-dense structures suggestive of lipid bodies (Figure 5D), nuclear membrane detachment, an increase in chromatin condensation, and nuclear fragmentation (Figure 5B). 

### 3.4. In Vitro Efficacy of Morolic Acid on Infection of J774A.1 Macrophages by L. (L.) amazonensis 

**Figure 6:**
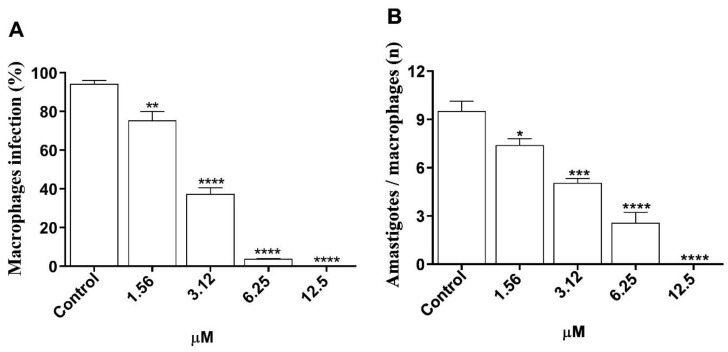
Antileishmanial activity of morolic acid against intramacrophagic amastigote forms after 72 h of exposure. J774A.1 macrophages (1 × 10^5^) were infected with promastigote forms in ratio of 10:1 macrophage and treated with morolic acid at 5% CO_2_ and 37 °C for 72 h. (**A**) percentage of infection and (**B**) number of amastigotes per macrophage. Results represent mean ± standard error of three independent experiments performed in triplicate. (*) *p* < 0.05 vs. control; (**) *p* < 0.01 vs. control; (***) *p* < 0.001 vs. control; (****) *p* < 0.0001 vs. control.

The values obtained from the morolic acid treatment of macrophages infected with *L.* (*L.*) *amazonensis* are presented using the percentage of infected macrophages (Figure 6A) and the number of amastigotes/macrophages (Figure 6B). For the infected macrophage percentage, it was observed that morolic acid was able to decrease the macrophage infection rate at all concentrations tested, with 34%, 77.5%, and 93% reductions at respective concentrations of 1.56 μM, 3.12 μM, and 6.25 μM, reaching a 100% reduction in infection rate at the concentration of 12.5 μM, all as compared to the negative control (Figure 6A). The second criterion evaluated was the number of amastigotes per infected macrophage. Treatment resulted in a significant reduction in the number of internalized amastigotes on the order of 59.1%, 71.6%, and 86.8% at respective concentrations of 1.56 μM, 3.12 μM, and 6.25 μM. At the highest concentration, 12.5 μM, a 100% reduction was observed as compared to the negative control (Figure 6).

### 3.5. Induction of Reactive Oxygen Species (ROS) and Nitric Oxide (NO) Production by Morolic Acid

**Figure 7:**
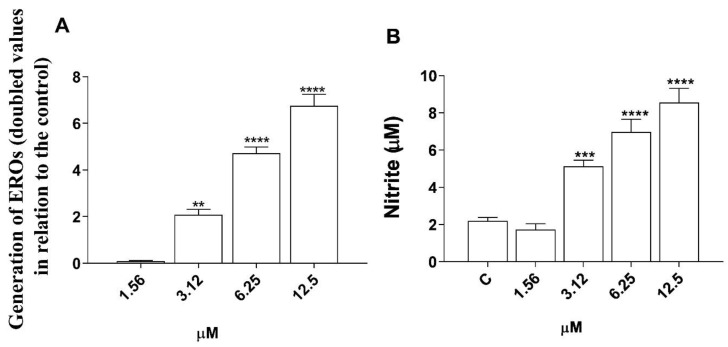
Morolic acid-induced changes in cellular mechanisms of J774A.1 macrophages. Changes in cellular mechanisms of J774A.1 macrophages. ROS (**A**) and NO (**B**) levels were measured in J774A.1 macrophages treated with morolic acid for 72 h at 37 °C and 5% CO_2_. Results represent mean ± standard error of three independent experiments performed in triplicate. (**) *p* < 0.01 vs. control; (***) *p* < 0.001 vs. control; (****) *p* < 0.0001 vs. control.

The results for the ROS and NO assay are plotted in Figure 7. We observed an increase in the levels of ROS and NO produced in macrophages infected and treated with morolic acid at its three highest concentrations (3.12 μM, 6.25 μM, and 12.5 μM,) compared to the negative control.

**Figure 8:**
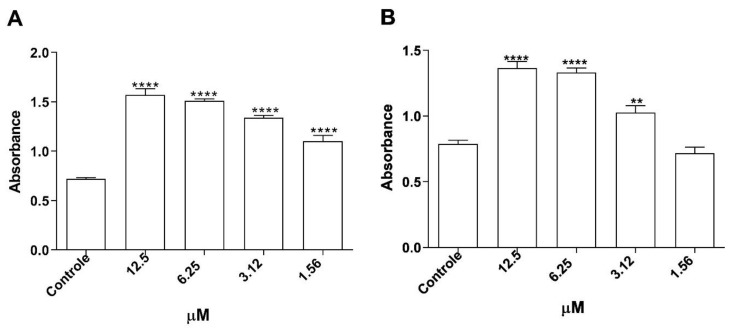
Changes in structural mechanisms of J774A.1 macrophages treated with morolic acid. J774A.1 macrophages were treated with serial concentrations for 72 h. Phagocytosis was analyzed by incorporation of zymosan stained with neutral red, solubilized by extraction solution. Lysosomal activity was analyzed by increase in neutral red (NR) retention after solubilization with extraction solution. Phagocytic capacity (**A**) and lysosomal volume (**B**). Results represent mean ± standard error of three independent experiments performed in triplicate. (**) *p* < 0.01 vs. control; (****) *p* < 0.0001 vs. control.

We observed that the substance increased lysosomal activity at concentrations of 12.5 μM, 6.25 μM, and 3.12 μM, relative to the negative control (Figure 8A). As to the phagocytic capacity for zymosan, we noticed a significant increase in phagocytosis at concentrations of 12.5 μM, 6.25 μM, and 3.12 μM compared to the negative control (Figure 8B).

### 3.6. Assessment of Cytokine Production in Infected Macrophages

**Figure 9:**
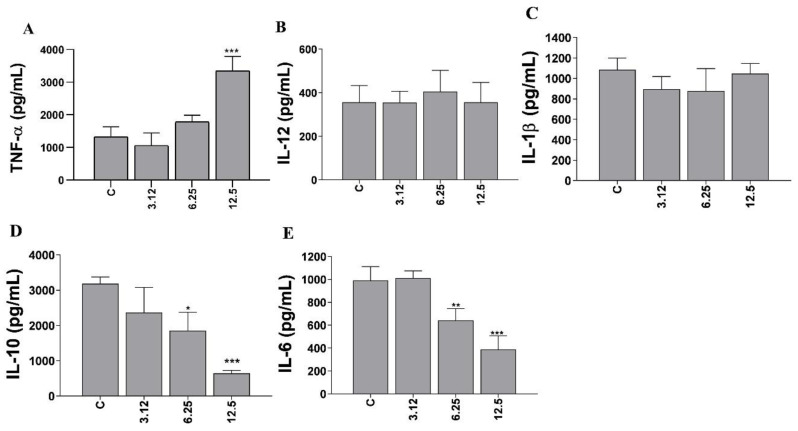
Levels of cytokines expressed by infected macrophages treated with morolic acid. Levels of cytokines TNF-α (**A**), IL-12 (**B**), IL-1β (**C**), IL-10 (**D**), and IL-6 (**E**) produced by uninfected and *Leishmania* (*Leishmania*) *amazonensis*-infected macrophages treated with morolic acid at 5% CO_2_ and 37 °C for 72 h. Results represent mean ± standard error of three independent experiments performed in triplicate. (*) *p* < 0.05 vs. control (c); (**) *p* < 0.01 vs. control (c); (***) *p* < 0.001 vs. control (c).

As illustrated in Figure 9, the application of morolic acid incited a protective cytokine reaction in the host, characterized by a significant increase (at the higher concentration), in the production of TNF-α (Figure 9A). This response was accompanied by a significant reduction (at the highest concentrations) in IL-10 levels (Figure 9D) and IL-6 levels (Figure 9E) in *L.* (*L.*) *amazonensis*-infected J774A.1 macrophages. Morolic acid presented no effect on IL-12 (Figure 9B) and IL-1α (Figure 9C) levels under the conditions evaluated.

## 4. Discussion

The therapeutic options currently available for leishmaniasis are limited, have significant costs, and present high toxicities with long treatment periods. These factors lead to low patient adhesion, increasing therapeutic failure, and the resistance observed in numerous parasite strains around the world. There is an urgent need for research involving new drugs for use against this group of diseases [28]. Natural plant compounds have proven to be a good source of safe and effective molecules for the treatment of leishmaniasis. Morolic acid is a secondary oleanane metabolite of the triterpene class (C_30_H_48_), which can be found in plants such as *Malus domestica* Borkh [29], *Phoradendron brachystachyum* Rizz [30], *Basil basilicum* Lesley, *Origanum vulgare* L. [31], and *Dalbergia ecastophyllum* (L.) Taub. [32]. These last three present proven leishmanicidal activity [33,34,35]. 

Morolic acid presents biological activities as an antimicrobial, anti-inflammatory, and antioxidant [36], but its anti-leishmania activity had not yet been investigated. Initially, it was observed that morolic acid was effective in inhibiting the growth of *L.* (*L.*) *amazonensis* promastigote forms from axenic amastigotes, with low IC_50_ values. The antileishmanial activity found for the compound is more pronounced than others triterpenes reported in the literature as potential leishmanicidal agents, such as ursolic acid, with an IC_50_ of 14.1 µg/mL for promastigotes and 2.24 µg/mL for axenic amastigotes of *L.* (*L.*) *amazonensis* [37], and Lupeol, with an IC_50_ value of 39.06 μg/mL for promastigotes and 44.1 µg/mL for amastigote forms of *L.* (*L.*) *amazonensis* [38]. Structural differences between triterpenes can determine the potency of the antileishmanial effect. Studies have shown that triterpenes presenting carboxyl groups at positions C-3 or C-28 have better leishmanicidal activity. This has been observed for ursolic acid, lupeol, betulin, and betulinic acid [16,39].

The data suggest that the promising activity of morolic acid may be related to the presence of a carboxyl group at position C-28. In infections caused by parasites of the *Leishmania* genus, the main targets are cells of the monocytic phagocytic system, mainly macrophages [40]. However, both the first- and second-line drugs currently used are toxic and non-selective, causing damage to the parasite and various host cells [8]. Drugs that may be cytotoxic to, alter, or damage the host cell are not promising.

When evaluating the safety of morolic acid in J774A.1 macrophages and VERO CCL 81, it was observed that the compound presents antileishmanial activity in non-toxic concentrations, generating a high SI. According to Furtado (2017) [41], for a compound to be safe its SI must be greater than 20; this was found for morolic acid, making its antileishmanial activity even more promising. On the basis of its SI, morolic acid was shown to be 79.8 times more selective for promastigotes, 24.8 times more selective for axenic amastigotes of *L.* (*L.*) *amazonensis* than meglumine antimoniate, and 57.8 times more selective for promastigotes and 32.1 times more selective for axenic amastigotes of *L.* (*L.*) *amazonensis* than amphotericin B. Various studies corroborate our findings, revealing that triterpenes such as lupeol, betulin [16,42,43], and betulinic acid [44] also demonstrate low or no cytotoxic effects in different cell lines. This suggests using this class of molecules in antileishmanial therapeutic studies.

In a model that most closely resembles in vivo efficacy, experiments were performed in concentrations capable of safely inhibiting the growth of intramacrophagic amastigote parasites [6]. Morolic acid significantly reduced macrophage infection and the number of intramacrophagic amastigotes. This activity might be explained by a double mode of action, in which morolic acid not only acts directly on the parasite but also activates an immunomodulatory response that increases the microbicidal power of the macrophages through mechanisms such as phagocytosis, lysosomal volume increases, and other cellular mechanisms, including NO and ROS increases [22,37]. These mechanisms were also evaluated in this work.

In leishmaniasis, resistance to infection has been associated with the development of Th1 immune response, with notable amounts of the cytokine interferon gamma (IFN-γ) that are produced mainly by NK and T cells [45]. As phagocytes are exposed to IFN-γ, the classic activation and induction of microbicidal mechanisms occur, with the participation of the enzyme inducible nitric oxide synthase (iNOS), which synthesizes NO, a mediator of inflammation. Together, other ROS and reactive nitrogen control the parasite load. Natural compounds capable of increasing the Th1 response can help fight the parasite [46]. In the present study, it was observed that morolic acid increases ROS and NO production in macrophages infected by *L.* (*L.*) *amazonensis* at the same concentrations in which the macrophage parasite load decreases, suggesting the participation of these mechanisms in the observed antileishmanial activity.

In addition to molecular mechanisms, structural mechanisms such as phagocytosis and lysosomal activity play a fundamental role in the response against invading organisms [22]. When phagocytosis and lysosomes are activated in the innate immune response, we note the increased internalization of the pathogen and its degradation through acid hydrolases and ROS produced in lysosomal vacuoles, with a consequent presentation of peptides derived from antigens [27,38]. The studied compound proved to be effective in activating these macrophage mechanisms, resulting in an increase in the lysosomal volume and phagocytic capacity of cells at all concentrations tested.

To observe the principal morphological changes caused by morolic acid, promastigote forms of *L.* (*L.*) *amazonensis* were analyzed using both SEM and TEM. Using SEM, extracellular evaluations of the parasite were performed, which presented structural changes, shortening, and wrinkling of the plasma membrane. The emergence of morphological changes such as a loss of membrane integrity is suggestive of death by apoptosis [47]. TEM assays were used to confirm programmed cell death through an analysis of intracellular parasite structures. Besides noting intracellular disorganization and altered morphology, there was also an increase in cytoplasmic vacuoles and the appearance of lipid bodies. Nuclear damage was observed, resulting in the displacement of the nuclear membrane and nuclear fragmentation, with loss of contents. According to Vannier-Santos and Castro [48], in treated parasites, the emergence of nuclear changes is associated with apoptosis, since changes in nuclear morphology can be considered an indicator of programmed cell death.

Studies have already demonstrated that parasites treated with ursolic acid and lupeol present significant intracellular changes. These compounds are able to inhibit mitochondrial transmembrane potential and damage the nucleus and cytoplasmic organelles in *L.* (*L.*) *infantum;* this suggests death by apoptosis [20]. Therefore, morphological changes related to programmed death cell in *L.* (*L.*) *amazonensis,* such as damage to the plasma and nuclear membranes or fragmentation of the nucleus and intracellular compartments, may be linked to triterpene antileishmanial activity. 

When performing analyses on the decreased macrophage infection rate, we verified more favorable results for the intramacrophagic amastigote form than for the promastigote form and axenic amastigotes. This finding required the investigation of the hypothesis that the antileishmanial effect of the substance could be intrinsically linked to immunomodulatory activity. Correlating with the immunomodulatory response, morolic acid stimulated the production of TNF-α in infected and treated macrophages; this corroborates the hypothesis that the compound can stimulate the Th1 pathway, which leads to combating the parasite. However, morolic acid could not significantly reduce the production of IL-12.

There are two main mechanisms that trigger macrophage activation during the innate immune response to *Leishmania* sp. First, Natural Killer cells (NK) play a crucial role in generating interferon-gamma (INF-γ). Secondly, the activation of Toll-like receptors (TLRs) has been noted, which triggers the production of tumor necrosis factor alpha (TNF-α). This second process is directly associated with macrophage activation, which aims to effectively eliminate the parasite [45]. In studies performed by Jesus et al., 2021 [16], triterpenes demonstrated the ability to modulate the immune response, directing it confluently towards a Th1-type orientation, which corroborates the results found for morolic acid. It is crucial to highlight that in addition to its intrinsic effectiveness in combating parasites, the therapeutic activity exerted by this class of compounds includes inducing a greater immune response.

Unlike the phenomenon of resistance, susceptibility to leishmaniasis is concomitantly characterized by the amplification of interleukin-10 (IL-10) levels, a cytokine molecule recognized for its anti-inflammatory activity and ability to exert inhibitory effects on the Th1-type immune response. In this context, an increase in IL-10 levels often leads to the expansion of the parasitic infection population, in addition to contributing to the establishment of a chronic state of infection [16]. As to the association of the Th2 profile with cytokines such as IL-10 and IL-6, it was observed that morolic acid in the highest concentrations tested exhibited a remarkable ability to significantly suppress the production of these cytokines compared to the untreated control group. This inhibitory effect on IL-10 and IL-6 synthesis results in benefits for the host, since it helps to regulate these immune system mediators and consequently promote the fight against the parasite [45]. These observations suggest the likely beneficial influence of morolic acid in providing a favorable immune response against *L.* (*L.*) *amazonensis* infection in infected macrophages through the promotion of increases in pro-inflammatory cytokines, as well as decreases in anti-inflammatory cytokines.

## 5. Conclusions

Based on these results, it can be inferred that morolic acid was selective and effective in controlling promastigote forms of *L. amazonensis* axenic amastigotes. This study also demonstrated that antileishmanial activity is correlated with immunomodulatory activity, which increases oxidative stress levels in macrophages responsible for infection control. The findings support the conclusion that morolic acid is a potential antileishmanial candidate and encourage future in vivo studies toward developing new leishmanicidal agents.

## Figures and Tables

**Table 1:** Anti-leishmania activity, cytotoxic effect against macrophages, and selectivity index (SI) values calculated for morolic acid, meglumine antimoniate, and amphotericin B.

	J774A.1	VERO CCL 81	Promastigotes			Axenic Amastigotes			Intracellular Amastigotes		
	CC_50_ µM	CC_50_ µM	IC_50_ µM	SI_J774A.1_	SI_VERO_	EC_50_ µM	SI_J774A.1_	SI_VERO_	EC_50_ µM	SI_J774A.1_	SI_VERO_
Morolic acid	68.61 ± 2.46	82.94 ± 1.84	1.13 ± 0.04	60.7	73.39	2.74 ± 0.03	25.04	30.27	2.56 ± 0.08	26.8	32.39
Amphotericin B	0.38 ± 0.06	0.32 ± 0.08	0.36 ± 0.03	1.05	0.9	0.23 ± 0.01	0.78	360.6	0.29 ± 0.04	1.31	1.10
Meglumine antimoniate	16433 ± 43.2	17363 ± 94.7	21564 ± 90.85	0.76	0.80	1017.4 ± 25.72	1.01	82.11	541 ± 13.5	30.37	32.09

SI = CC_50_/IC_50_ or EC_50_.

## Data Availability

The raw data supporting the conclusions of this article will be made available by the authors on request.
